# Prevalence and Risk Factors of Chronic Otitis Media: The Korean National Health and Nutrition Examination Survey 2010–2012

**DOI:** 10.1371/journal.pone.0125905

**Published:** 2015-05-15

**Authors:** Mina Park, Ji Sung Lee, Jun Ho Lee, Seung Ha Oh, Moo Kyun Park

**Affiliations:** 1 Department of Otorhinolaryngology-Head and Neck Surgery, Seoul National University College of Medicine, Seoul National University Hospital, Seoul, Republic of Korea; 2 Clinical Research Center, Asan Medical Center, Seoul, Republic of Korea; 3 Sensory Organ Research Institute, Seoul National University Medical Research Center, Seoul, Republic of Korea; Katholieke Universiteit Leuven, BELGIUM

## Abstract

**Background:**

The performance of nationwide studies of chronic otitis media (COM) in adults has been insufficient in Korea. We evaluated the prevalence and risk factors of COM in Korea.

**Methods:**

This study was conducted using data from the fifth Korean National Health and Nutrition Examination Survey (n = 23,621). After excluding the subjects under 20 year old and suffered from cancers, 16,063 patients were evaluated for COM. Participants underwent a medical interview, physical examination, endoscopic examination, and blood and urine test. COM was diagnosed by trained residents in the Department of Otorhinolaryngology using an ear, nose, and throat questionnaire and otoendoscopy findings. Data on the presence and absence of COM were collected. Multivariate logistic regression analyses were performed to identify its risk factors.

**Results:**

Of the 16,063 participants aged above 20 year old, the weighted prevalence of COM was 3.8%. In the multivariate analyses, the following factors showed high odds ratios (ORs) for COM: pulmonary tuberculosis (adjusted OR, 1.78; 95% confidence interval [CI], 1.06-3.01), chronic rhinosinusitis (adjusted OR, 1.87; 95% CI, 1.17-2.98), mild hearing impairment (adjusted OR, 1.95; 95% CI, 1.34-2.85), moderate hearing impairment (adjusted OR, 4.00; 95% CI, 2.21-7.22), tinnitus (adjusted OR, 1.82; 95% CI, 1.34-2.49), increased hearing thresholds in pure tone audiometry in the right ear (adjusted OR, 1.02; 95% CI, 1.01-1.03), and left ear (adjusted OR, 1.03; 95% CI, 1.02-1.04). The following factors showed low odds ratios for COM: hepatitis B (adjusted OR, 0.28; 95% CI, 0.08-0.94) and rhinitis (adjusted OR, 0.60; 95% CI, 0.42-0.88). In addition, high levels of vitamin D, lead, and cadmium, EQ-5D index; and low red blood cell counts were associated with development of COM (Student’s *t*-test, *P* < 0.01).

**Conclusions:**

Our population-based study showed that COM is not rare in Korea, and its development may be associated with various host and environmental factors. Further research on its relationships and the pathogenesis are needed.

## Introduction

Chronic otitis media (COM) is characterized by inflammation of the middle ear that results in long-term or permanent changes in the tympanic membrane. These changes include perforation, atelectasis, retraction, tympanosclerosis, and cholesteatoma. COM can be classified based on whether it involves active inflammation or is associated with a cholesteatoma [1]. This disorder is a major cause of acquired hearing loss, especially in developing countries, and is a major disease entity in the field of otolaryngology. It often requires expensive treatment and ear surgery, and can induce severe or fatal complications such as mastoiditis, facial nerve palsy, labyrinthitis, petrositis, brain abscessation, meningitis, and thrombophlebitis. COM also decreases patients’ quality of life. Chronic active or suppurative otitis media affects 65 to 330 million people worldwide, and more than half of these patients have significant hearing impairment. Worldwide, COM is responsible for an estimated 28,000 deaths annually, and is associated with a disease burden involving more than 2 million individuals daily [2].

Many previous studies have investigated the prevalence and risk factors of COM. Its reported prevalence in Southeast Asia, Africa, and Western Pacific countries is 2–4%, and that in North America and European countries is < 2%. Risk factors of COM include low socioeconomic status, malnutrition, high number of children in the household, family history, and passive exposure to smoking [3]. However, most studies have involved children, and have confined the study of otitis media to chronic suppurative otitis media, acute otitis media, or otitis media with effusion [4–13]. Moreover, the effects of various host and environmental factors have not been well defined. Information on the risk factors of COM would contribute to effective treatment and control of this disease.

Here, we identified the prevalence and risk factors of COM in Korea through analysis of data collected by medical interviews, endoscopic examinations, pure tone audiometry, and blood tests that included heavy metal levels.

## Materials and Methods

### Population

This study used the data of the fifth Korean National Health and Nutrition Examination Survey (KNHANES). This survey collected information, such as health and nutritional status, from a representative sample of the general Korean population to monitor the prevalence and control, and to reveal the risk factors, of certain chronic diseases. The KNHANES included information on the presence and absence of COM, which was diagnosed by otolaryngology residents.

In total, 23,621 individuals (8,313 in 2010, 7,887 in 2011, 7,421 in 2012) agreed to participate in the health surveys and underwent medical checkups that included ear, nose, and throat (ENT) examinations. Patients aged < 20 years (n = 5,744), those with cancer (n = 641), and those with a missed COM diagnosis (those in whom otoendoscopy was not performed because of the individual’s refusal or missed examination) (n = 1,173) were excluded. Finally, 16,063 individuals were analyzed in this study. The average patient age was 50.2 ± 16.3 years (range, 20–97 years), and the male: female ratio 1.00:1.35. Written informed consent was obtained from all of the participants prior to the survey. This study was approved by the Institutional Review Board of the Seoul National University Hospital (1409–079–609).

### ENT evaluation, medical history, and clinical examination

The diagnosis of COM was determined by trained residents using a systematic ENT questionnaire and the following otoendoscopy findings: tympanic membrane perforation and/or cholesteatoma, including congenital cholesteatoma, and a retraction pocket and/or otitis media with effusion, including patients with insertion of a ventilation tube. Because it is not easy to diagnose congenital cholesteatoma in adults and there is some debate about the definition of congenital cholesteatoma, we did not differentiate between cholesteatoma and congenital cholesteatoma in this study.

The prevalence of COM was analyzed in six different age groups, each covering a 10-year period, as well as between male and female patients.

Information on the patients’ socioeconomic status was investigated, including education level (less than middle school or beyond high school), income (< 25%, 25–50%, 50–75%, or > 75% according to the equivalized household income per month), occupation (white-collar: manager, professional, clerk, service/sales worker, unemployed, retired, student, or housewife; blue-collar: agriculture, forestry, fishery worker, craft and related trade worker, plant or machine operator or assembler, or simple laborer), residency (urban or rural area in accordance with the patient’s official address), and exposure to noise (earphone use in noisy situations or temporary exposure to noise). Information was also collected on each patient’s smoking status (nonsmoker, < 5 packs in their life; smoker, > 5 packs and currently smoking), alcohol drinking status (no, does not drink; yes, alcohol consumption one or more times per month during the past year), number of household members (1–2, 3–4, 5–6, or ≥ 7), and subjective health status (very good, good, average, poor, or very poor). Each patient’s body mass index was categorized as either < 25 or > 25 kg/m^2^.

Professional interviewers from the Korea Centers for Disease Control and Prevention provided a documented questionnaire and obtained the patients’ medical histories. Allergic rhinitis was diagnosed when patients had experienced symptoms of sneezing, rhinorrhea, nasal obstruction, and itching for the past year. The diagnosis of chronic rhinosinusitis was made when nasal polyps were observed during endoscopy or when more than one of the following symptoms was present: anterior/posterior nasal drip, nasal obstruction, facial pain/tenderness, and olfactory dysfunction for more than 3 months (anterior/posterior nasal drip or nasal obstruction should be included as a presenting symptom). Septal deviation was defined as asymmetrical displacement of the nasal septum to the left or right side of the nasal cavity after vasoconstriction of the nasal mucosa.

For otologic investigation, the patients were asked about their hearing and any symptoms of tinnitus by questionnaire, and physical examinations were conducted by the residents to assess the presence or absence of facial palsy and preauricular sinuses. In addition, pure tone audiometry was performed and calculated by averaging thresholds at 500 Hz, 1000 Hz, 2000 Hz, and 3000 Hz.

Blood samples were collected and analyzed in a single laboratory (Neodin Medical Institute, Seoul, Korea). The Euro Qol-5D (EQ-5D) is a standard tool used to measure patients’ health status in five dimensions: mobility, self-care, usual activities, pain/discomfort, and anxiety/depression [14,15]. Each dimension has three grades of severity: no problems (score of 1), moderate problems (score of 2), or serious problems (score of 3). The EQ-5D index is calculated from the EQ-5D score by applying a formula that assigns weights to each of the grades in each dimension. This formula differs among nations based on the value of the EQ-5D from population samples [16]. The KNHANES algorithm for calculating the EQ-5D index was applied in the present study; it ranged from 1 (best health) through 0 (equivalent to death) to—0.171 (worse than death).

### Statistical analysis

The data were analyzed with SAS software (version 9.4; SAS Institute Inc., Cary, NC, USA), which incorporates sample weights, and adjusts the analysis for the complex sample design of the survey. We used the KNHANES sampling weight variables along with a masked variance primary sampling unit and stratum variables. This adjustment allowed for extrapolation from the samples to the noninstitutionalized civilian Korean population as a whole. The survey sample weights were used in all of the analyses. Statistics were used to describe the general characteristics, medical conditions, otologic conditions, blood test results, and questionnaire results regarding quality of life of all of the samples according to COM. Data were tested for statistical significance by applying the chi-squared test for categorical variables and the Student’s *t*-test for continuous variables.

Logistic regression analysis was performed to identify risk factors independently associated with COM. Multiple logistic regression analyses were performed and included variables with P values < 0.2 in the univariate analysis to estimate their adjusted odds ratios (ORs) and 95% confidence intervals (CIs). However, the blood levels of heavy metals were excluded from the logistic regression analysis because the fifth KNHANES investigated these in few participants. A *P* value < 0.05 indicated statistical significance.

## Results

### Prevalence of COM

Among the 16,063 participants, the weighted prevalence of COM was 3.8% (tympanic perforation, 2.17%; cholesteatoma, 1.82%; otitis media with effusion, 0.68%), and those of the right, left, and both ears were 1.5%, 1.4%, and 0.9%, respectively.

**Fig 1:**
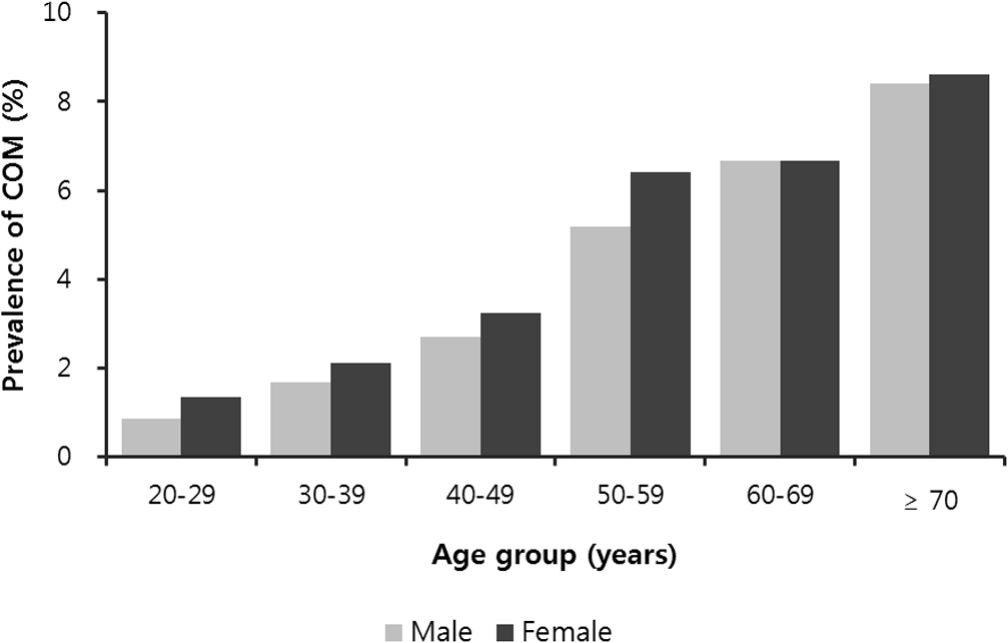
Prevalence of chronic otitis media by age group and sex in Korean adults. The prevalence of chronic otitis media increased with age in both sexes.

The prevalence of COM according to the general characteristics of the participants is described in Table 1. Age, sex, education, residence, earphone use in noisy situations, number of household members, and subjective health status affected the prevalence of COM. An increased prevalence was associated with old age (*P* < 0.0001), female sex (*P* = 0.0287), lower education level (*P* < 0.0001), urban residence (*P* = 0.0239), not using earphones in noisy situations (*P* < 0.0001), fewer household members (*P* < 0.0001), and a poor subjective health status (*P* < 0.0001). There were no significant differences between male and female patients among the six different age groups, but the prevalence of COM tended to increase with age (Fig 1).

**Table 1:** Prevalence of chronic otitis media according to the general characteristics of KNHNES participants.

		COM weighted*, % (SE)		
Characteristics	Unweighted total number	No	Yes	P value
**Overall**	16063	96.2 (0.2)	3.8 (0.2)	
**Affected ear**				
Right		98.5 (0.1)	1.5 (0.1)	
Left		98.6 (0.1)	1.4 (0.1)	
Both		99.1 (0.1)	0.9 (0.1)	
**Age(year)**				<0.0001
**20–29**	1797	98.9 (0.3)	1.1 (0.3)	
**30–39**	3154	98.1 (0.3)	1.9 (0.3)	
**40–49**	2989	97.0 (0.4)	3.0 (0.4)	
**50–59**	3105	94.2 (0.6)	5.8 (0.6)	
**60–69**	2632	93.3 (0.6)	6.7 (0.6)	
**≥70**	2386	91.5 (0.7)	8.5 (0.7)	
**Sex**				0.0287
**Male**	6830	96.7 (0.3)	3.3 (0.3)	
**female**	9233	95.8 (0.3)	4.2 (0.3)	
**Education**				<0.0001
**≤Middle school**	5492	93.1 (0.4)	6.9 (0.4)	
**≥High school**	10140	97.6 (0.2)	2.4 (0.2)	
**Missing**	431			
**Income**				0.1732
**<25%**	3891	96.0 (0.4)	4.0 (0.4)	
**25–50%**	3959	96.5 (0.3)	3.5 (0.3)	
**50–75%**	4008	95.8 (0.4)	4.2 (0.4)	
**≥75%**	4012	96.8 (0.4)	3.2 (0.4)	
**missing**	193			
**Occupation**				0.7594
**white collar**	12767	96.4 (0.2)	3.6 (0.2)	
**blue collar**	2828	96.2 (0.4)	3.8 (0.4)	
**Residence**				0.0239
**Rural**	12780	96.5 (0.2)	3.5 (0.2)	
**Urban**	3283	95.4 (0.4)	4.6 (0.4)	
**Body mass index(kg/m2)**				0.3783
**<25**	10857	96.1 (0.2)	3.9 (0.2)	
**≥25**	5151	96.5 (0.3)	3.5 (0.3)	
**Missing**	55			
**Noise**				
**Earphone use in noisy situation**				<0.0001
**Yes**	1376	98.4 (0.4)	1.6 (0.4)	
**No**	14627	96.0 (0.2)	4.0 (0.2)	
**Missing**	60			
**Temporary exposure to noise**				0.1384
**Yes**	3347	96.7 (0.4)	3.3 (0.4)	
**No**	12643	96.1 (0.2)	3.9 (0.2)	
**Smoking status**				0.0605
**No**	12398	96.1 (0.2)	3.9 (0.2)	
**Yes**	3245	96.9 (0.4)	3.1 (0.4)	
**Missing**	420			
**Alcohol consumption**				0.0681
**No**	7242	95.9 (0.3)	4.1 (0.3)	
**Yes**	8335	96.6 (0.2)	3.4 (0.2)	
**Missing**	486			
**Household members(persons)**				<0.0001
**1–2**	5234	94.2 (0.4)	5.8 (0.4)	
**3–4**	8609	97.1 (0.2)	2.9 (0.2)	
**5–6**	2052	96.7 (0.5)	3.3 (0.5)	
**≥7**	146	98.1 (1.0)	1.9 (1.0)	
**Missing**	22			
**Subjective health status**				<0.0001
**Very good**	703	97.2 (0.7)	2.8 (0.7)	
**Good**	4560	97.1 (0.3)	2.9 (0.3)	
**Average**	7491	96.5 (0.3)	3.5 (0.3)	
**Poor**	2413	94.7 (0.6)	5.3 (0.6)	
**Very poor**	490	91.9 (1.3)	8.1 (1.3)	
**Missing**	406			

COM, chronic otitis media; SE, standard error; KNHANES, Korean national health and nutrition examination survey;

*weighted for the multistage sampling design of KNHANES 2010~2012

Table 2 shows that diverse medical conditions were associated with COM. Hypertension and diabetes mellitus were associated with an increased prevalence of COM. In contrast, hepatitis B and allergic rhinitis were associated with decreased prevalence of COM.

**Table 2:** Prevalence of chronic otitis media according to the medical conditions of KNHANES participants.

		COM weighted*, % (SE)		
Characteristics	Unweighted total number	No	Yes	P value
**Hypertension**				<.0001
No	10742	97.2 (0.2)	2.8 (0.2)	
Yes	4744	93.8 (0.5)	6.2 (0.5)	
**Diabetes mellitus**				0.0001
No	13228	96.6 (0.2)	3.4 (0.2)	
Yes	1460	93.1 (0.9)	6.9 (0.9)	
**Pulmonary Tuberculosis**				0.1219
No	14903	96.4 (0.2)	3.6 (0.2)	
Yes	750	94.7 (1.1)	5.3 (1.1)	
**Hepatitis B**				0.0004
No	15414	96.3 (0.2)	3.7 (0.2)	
Yes	237	98.6 (0.6)	1.4 (0.6)	
**Atopic dermatitis**				0.5667
No	15298	96.3 (0.2)	3.7 (0.2)	
Yes	353	97.2 (1.5)	2.8 (1.5)	
**Allergic rhinitis**				0.0019
No	12023	95.9 (0.2)	4.1 (0.2)	
Yes	4033	97.1 (0.3)	2.9 (0.3)	
**Chronic rhinosinusitis**				0.1657
No	14922	96.3 (0.2)	3.7 (0.2)	
Yes	928	95.1 (0.9)	4.9 (0.9)	
**Septal deviation**				0.2119
No	8155	96.5 (0.3)	3.5 (0.3)	
Yes	7701	96.0 (0.3)	4.0 (0.3)	

COM, chronic otitis media; SE, standard error; KNHANES, Korean national health and nutrition examination survey;

*weighted for the multistage sampling design of KNHANES 2010~2012

Several items relevant to otologic findings are described in Table 3. Subjective hearing discomfort and tinnitus were associated with COM. Several other items are shown in Table 4. Blood test analysis showed that COM was associated with decreased red blood cell counts (Student’s *t*-test, *P* < 0.0001) and increased levels of vitamin D, lead, and cadmium (Student’s t-test, *P* < 0.0172). In the pure tone audiometry examinations, COM was associated with an increased hearing threshold in both ears (Student’s *t*-test, *P* < 0.0001). With respect to health status, COM was associated with a high EQ-5D index (*P* < 0.0001).

**Table 3:** Prevalence of chronic otitis media according to otologic conditions of KNHANES participants.

		COM weighted*, % (SE)		
Characteristics	Unweighted total number	No	Yes	P value
**Subjective hearing**				<0.0001
Not discomfort	13715	97.5 (0.2)	2.5 (0.2)	
A little discomfort	1852	89.9 (1.0)	10.1 (1.0)	
A lot of discomfort	444	72.7 (2.9)	27.3 (2.9)	
Cannot hearing anything	45	76.0 (7.1)	24.0 (7.1)	
**Tinnitus**				<0.0001
Yes	3593	93.3 (0.5)	6.7 (0.5)	
No	12425	97.1 (0.2)	2.9 (0.2)	
Not remember	38	96.6 (2.4)	3.4 (2.4)	
**Facial palsy(House-brackman grade)**				0.9230
Ⅰ~Ⅱ	16027	96.2 (0.2)	3.8 (0.2)	
Right side Ⅲ~Ⅵ	21	95.6 (4.4)	4.4 (4.4)	
Left side Ⅲ~Ⅵ	15	94.3 (5.4)	5.7 (5.4)	
**Preauricular sinus, right**				0.3345
Normal	15919	96.3 (0.2)	3.7 (0.2)	
Abnormal	144	93.9 (2.4)	6.1 (2.4)	
**Preauricular sinus, left**				0.1339
Normal	15913	96.2 (0.2)	3.8 (0.2)	
Abnormal	150	97.7 (1.0)	2.3 (1.0)	

KNHANES, Korean national health and nutrition examination survey;

*weighted for the multistage sampling design of KNHANES 2010~2012

**Table 4:** Prevalence of chronic otitis media according to blood test, hearing threshold, and questionnare of quality of life.

		COM weighted* (SE)		
Variable	Unweighted total number	No	Yes	P value
**Blood test**				
White blood cells (Thous/μL)	15267	6.13 (0.02)	6.18 (0.08)	0.5260
Red blood cells (Mil/μL)	15267	4.64 (0.01)	4.53 (0.02)	<.0001
Platelet (Thous/μL)	15267	255.36 (0.64)	251.41 (2.63)	0.1511
Vitamin D (ng/mL)	15322	17.24 (0.13)	17.95 (0.32)	0.0172
**Heavy metals**				
Lead (μg/dL)	5420	2.301 (0.019)	2.659 (0.148)	0.0163
Mercury (μg/L)	5420	4.387 (0.070)	4.639 (0.255)	0.3416
Cadmium (μg/L)	5420	1.084 (0.012)	1.385 (0.061)	<.0001
Zinc (μg/dL)	1790	138.08 (1.79)	134.21 (5.33)	0.4436
**Average hearing threshold**				
Right ear (dB)	14983	13.7 (0.2)	30.9 (1.1)	<0.0001
Left ear (dB)	14982	13.9 (0.2)	32.9 (1.4)	<0.0001
**EQ-5D index**	15640	0.95 (0.00)	0.93 (0.01)	<.0001

EQ-5D, Euro Qol-5D; KNHANES, Korean national health and nutrition examination survey;

*weighted for the multistage sampling design of KNHANES 2010~2012

### Multivariate analyses of risk factors

The results of the multivariable-adjusted analyses between COM and several patient characteristics are shown in Table 5. A history of pulmonary tuberculosis (adjusted OR, 1.78; 95% CI, 1.06–3.01) and chronic rhinosinusitis (adjusted OR, 1.87; 95% CI, 1.17–2.98) were positively associated with COM. In contrast, hepatitis B (adjusted OR, 0.28; 95% CI, 0.08–0.94) and allergic rhinitis (adjusted OR, 0.60; 95% CI, 0.42–0.88) were negatively associated with COM. Among patients with no discomfort in subjective hearing, the risk of COM was significantly higher in those with slight discomfort (adjusted OR, 1.95; 95% CI, 1.34–2.85) and severe discomfort (adjusted OR, 4.00; 95% CI, 2.21–7.22). In addition, tinnitus was definitely associated with a higher risk of COM (adjusted OR, 1.82; 95% CI, 1.34–2.49). Compared to patients with normal hearing thresholds in pure tone audiometry, the ORs of COM with increased hearing thresholds were 1.02 and 1.03 in the right and left ear, respectively. With respect to health status, the EQ-5D index was positively associated with COM (adjusted OR, 6.25; 95% CI, 1.94–20.16).

**Table 5:** Adjusted odds ratio for the association between chronic otitis media and risk factors.

	Adjusted OR	95% CI	P value
**Age(year)**			
20–29	1.00		
30–39	1.25	(0.62–2.53)	0.5334
40–49	1.73	(0.75–3.97)	0.1974
50–59	1.64	(0.71–3.79)	0.2464
60–69	0.70	(0.28–1.75)	0.4422
≥70	0.65	(0.24–1.72)	0.3837
**Sex (female)**	1.44	(0.94–2.21)	0.0967
**Education (**≤Middle school)	1.06	(0.74–1.52)	0.7598
**Income**			
<25%	1.01	(0.68–1.49)	0.9750
25–50%	0.96	(0.63–1.45)	0.8299
50–75%	1.27	(0.85–1.90)	0.2375
≥75%	1.00		
**Residence (Urban)**	1.03	(0.74–1.41)	0.8820
**Earphone use in noisy situation**	0.94	(0.44–2.01)	0.8674
**Temporary exposure to noise**	1.00	(0.68–1.46)	0.9781
**Smoking status**	0.93	(0.62–1.40)	0.7266
**Alcohol consumption**	1.36	(1.00–1.87)	0.0530
**Household members** (persons)			
1–2	1.42	(0.37–5.47)	0.6131
3–4	0.91	(0.24–3.43)	0.8936
5–6	1.19	(0.29–4.84)	0.8085
≥7	1.00		
**Subjective health status**			
Very good	1.00		
Good	1.23	(0.60–2.55)	0.5745
Average	1.14	(0.52–2.49)	0.7519
Poor	1.45	(0.63–3.33)	0.3789
Very poor	1.60	(0.67–3.86)	0.2916
**Hypertension**	1.27	(0.95–1.69)	0.1086
**Diabetes mellitus**	1.31	(0.89–1.94)	0.1681
**Pulmonary tuberculosis**	1.78	(1.06–3.01)	0.0302
**Hepatitis B**	0.28	(0.08–0.94)	0.0395
**Allergic rhinitis**	0.60	(0.42–0.88)	0.0079
**Chronic rhinosinusitis**	1.87	(1.17–2.98)	0.0087
**Subjective hearing**			
Not discomfort	1.00		
A little discomfort	1.95	(1.34–2.85)	0.0005
A lot of discomfort	4.00	(2.21–7.22)	<.0001
Cannot hearing anything	2.64	(0.53–13.04)	0.2336
**Tinnitus**			
Yes	1.82	(1.34–2.49)	0.0002
No	1.00		
Not remember	1.45	(0.23–9.03)	0.6882
**Preauricular sinus, left**	0.61	(0.17–2.16)	0.4448
**Average hearing threshold**			
Right ear (dB)	1.02	(1.01–1.03)	0.0016
Left (dB)	1.03	(1.02–1.04)	<.0001
**Blood test**			
Red blood cells	0.93	(0.62–1.42)	0.7473
Platelet	1.00	(1.00–1.00)	0.9851
Vitamin D (ng/mL)	1.00	(0.98–1.02)	0.9018
**EQ-5D index**	6.25	(1.94–20.16)	0.0022

OR, odds ratio; CI, confidence interval; EQ-5D, Euro Qol-5D

## Discussion

To the best of our knowledge, this is the first community-based study to examine the prevalence and risk factors of COM by assessment of clinical examination and laboratory test results among the Korean population. In addition, this study investigated the relationship between COM and the results of several blood tests, including blood levels of heavy metals. We believe that this study accurately represents the data of the Korean population, because the data from the KNHANES are comprehensive, statistically verified, and nationally representative [17].

The weighted prevalence of COM in Korean adults aged > 20 years was 3.8%. Generally, the prevalence of COM has been gradually declining worldwide. Many previous studies from several countries have reported that the prevalence of COM has been gradually declining on an annual basis. This decline has resulted from the wide use of antibiotics [18], improved nutrition and hygiene statuses secondary to economical growth, improved public welfare (i.e., coverage of universal health in Korea in 1989), and easy access to medical centers. Although the prevalence of COM is very diverse among different countries, our data are similar to those of East Asia (3.67%) according to the estimation released by the World Health Organization in 2004 [2,19]. Kim et al. [20,21] reported that the overall prevalence of otitis media in all of the age groups in Korea was 4.59% in 1981 and 2.85% in 1991, according to the results of nationwide surveys. Their study included both COM and otitis media with effusion. The prevalence of COM was higher in this study than in 1991. However, because the 1991 study was conducted for only 3 months (from July to October), the data may have been influenced by the season. Moreover, in the 1991 study, otorhinolaryngologists visited the subjects’ houses door to door and examined the tympanic membrane using an otoscope. In the present study, the participants visited the clinic and were examined with endoscopes by trained ENT residents. Therefore, an appropriate interpretation may be that an increase occurred in the rate of diagnosis of COM, and not the prevalence of COM.

We found high ORs for COM in relation to the following factors: hypertension, diabetes mellitus, pulmonary tuberculosis, chronic rhinosinusitis, mild/moderate hearing impairment, tinnitus, and increased hearing thresholds in pure tone audiometry. Many previous studies have shown that diabetes and cardiometabolic disease are associated with hearing loss [22–25], depending on the process of diabetic microangioapathy and macroangiopathy [23,26,27]. Moreover, otitis media is prevalent among people with high blood pressure and diabetes mellitus. Our result is in accordance with this study [28]. No previous studies have shown an association between pulmonary tuberculosis and COM. The association found in our study seems to reflect the special situation in Korea. Korea is endemic for pulmonary tuberculosis. Pulmonary tuberculosis might aggravate the patient’s general condition, which may be associated with the increased OR. The association between chronic rhinosinusitis and otitis media is well documented [29], and the findings in the present study are in accordance with previously established data. The polyps and swollen mucosa in patients with rhinosinusitis obstruct the eustachian tube orifice, leading to eustachian tube dysfunction. This is supported by the fact that the nasal cavity and eustachian tube share the same bacteria [29]. COM induces either conductive or sensorineural hearing loss. Our data also showed an association between subjective/objective hearing discomfort and COM, and also between tinnitus and COM.

In the present study, the ORs for COM were low in relation to hepatitis B and allergic rhinitis. Regarding hepatitis B, no previously published studies or other scientific data have reported this relationship. Further studies will be needed to investigate the cause of the relationship between hepatitis B and COM. Allergic rhinitis is a widely accepted risk factor for otitis media with effusion [30–34], but not for chronic suppurative otitis media [35]. The present study showed that allergic rhinitis was negatively associated with COM, although the precise mechanism underlying this association remains unclear. This study included more patients with chronic suppurative otitis media than with otitis media with effusion, because we only included adults > 20 years of age.

Most studies have suggested that otitis media has a negative effect on patients’ quality of life [36,37]. In the present study, quality of life showed a strong positive association with the prevalence of COM. Considering that one of the etiologies of COM is infection, and that infection is negatively associated with quality of life, our result opposes the commonly belief. This implies that the etiologies of COM are multifactorial, and that many critical factors other than infections influence COM. Interestingly, we found an association between heavy metals and COM. To the best of our knowledge, there have been no previous reports on this association. Although the reason for this association is unclear, it seems that high blood levels of heavy metals may destroy the middle ear mucosa or prevent the recovery of the tympanic membrane. However, these data were excluded from the multivariate analysis due to the reliability of the data because blood tests, including heavy metals, were only conducted in some cases.

This study had some limitations. First, COM is a generic term for all forms of middle ear inflammation. That is, it does not distinguish otitis media from effusion, cholesteatoma, and chronic suppurative otitis media. These conditions share some aspects of their pathogenesis, but differ with respect to other aspects. Second, because this was a cross-sectional study, the causative relationships between risk factors and COM may be difficult to determine. Third, an association between gastroesophageal reflux and otitis media in adults has not been proposed [38]. Gastric acid, biliary acid, and pepsin are refluxed into the esophagus by relaxation of the lower esophageal sphincter, and subsequently reach the nasopharynx, eustachian tube, and middle ear [39]. Although the casual link between gastroesophageal reflux and COM is not definitive, it might be clinically worthwhile to evaluate this relationship in the Korean population. If a sixth nationwide study can compensate for the weak points of the fifth study, solid data could be collected.

In conclusion, this population-based study revealed that 3.8% of the Korean population has COM. Various host factors, including chronic rhinosinusitis, hearing impairment, and tinnitus, were associated with risk of COM. Some environmental factors, such as high lead or cadmium, may also be associated with an increased risk of COM. Further studies are required to ascertain these associations.
